# Draft Genome Sequences of Heavy Metal-Resistant Listeria monocytogenes Strains of Sequence Type 14 (Clonal Complex 14) from Human Listeriosis Cases in Sweden

**DOI:** 10.1128/mra.00406-23

**Published:** 2023-07-10

**Authors:** Sangmi Lee, Wilhelm Tham, Marie-Louise Danielsson-Tham, Gloria Lopez-Valladares, Yi Chen, Phillip Brown, Sophia Kathariou

**Affiliations:** a Department of Food and Nutrition, Chungbuk National University, Cheongju, Chungbuk, South Korea; b School of Hospitality, Culinary Arts, and Meal Science, Örebro University, Örebro, Sweden; c Division of Microbiology, Center for Food Safety and Applied Nutrition, Food and Drug Administration, College Park, Maryland, USA; d Department of Plant and Microbial Biology, North Carolina State University, Raleigh, North Carolina, USA; e Department of Food, Bioprocessing, and Nutrition Sciences, North Carolina State University, Raleigh, North Carolina, USA; Queens College Department of Biology

## Abstract

Listeria monocytogenes of clonal complex 14 (CC14) is a potentially hypervirulent clone of serotype 1/2a but remains poorly characterized. We report the genome sequences of five sequence type 14 (ST14) (CC14) strains from human listeriosis cases in Sweden, which harbor a chromosomal heavy metal resistance island that is generally uncommon in serotype 1/2a.

## ANNOUNCEMENT

Listeria monocytogenes is a Gram-positive foodborne pathogen that causes listeriosis in humans and animals (1–3). At high risk are pregnant women and their fetuses, immunocompromised patients, and elderly individuals (1–3). Symptoms include septicemia, meningitis, and stillbirths, and mortality rates remain high (1–3). Serotypes 1/2a, 1/2b, and 4b contribute to most human listeriosis cases, and all leading hypervirulent clones belong to lineage I, serotype 4b (3, 4). However, the lineage II, serotype 1/2a clone of clonal complex 14 (CC14) appears to also have hypervirulence potential, based on data obtained with the Galleria mellonella model (5). Previously, chromosomal islands (*Listeria* genomic island 2 [LGI2] and LGI2-1) harboring heavy metal (cadmium and arsenic) resistance genes were found primarily in serotype 4b, especially strains of the hypervirulent CC1 and CC2, but were uncommon in serotypes 1/2a or 1/2b (6). We report the whole-genome sequences of five sequence type (ST) 14 (CC14) strains obtained from human listeriosis cases in Sweden in 2000 to 2008 (Table 1). All five strains were resistant to both cadmium and arsenic and harbored a chromosomal LGI2 that was highly similar to that of serotype 4b and was inserted in the *LMOf2365_0271* homolog, as previously observed in the ST14 (CC14) strain 2012-0070 from North Carolina, USA (6).

**TABLE 1:** Characteristics of the genomes of the five L. monocytogenes ST14 strains investigated in this study

Characteristic	Finding for strain:				
	CFSAN061434	CFSAN061444	CFSAN061464	CFSAN061476	CFSAN061478
Year/country of isolation	2000/Sweden	2001/Sweden	2003/Sweden	2007/Sweden	2008/Sweden
Source	Human (blood)	Human (blood)	Human (blood)	Human (blood)	Human (blood)
GenBank accession no.	ABKMVX000000000.1	ABKMVM000000000.1	ABKPJI000000000.1	ABKPKC000000000.1	ABKPJN000000000.1
BioProject accession no.	PRJNA215355	PRJNA215355	PRJNA215355	PRJNA215355	PRJNA215355
BioSample accession no.	SAMN33142786	SAMN33142993	SAMN33246784	SAMN33246678	SAMN33246780
Pathogen detection assembly accession no.	PDT001608011.1	PDT001608058.1	PDT001612391.1	PDT001612370.1	PDT001612387.1
Raw read accession no.	SRR23352938	SRR23353127	SRR23409844	SRR23409732	SRR23409840
Sequencing method	MiSeq	MiSeq	NextSeq 500	NextSeq 500	NextSeq 500
No. of read pairs	748,095	911,685	1,144,077	1,449,918	1,758,868
Total length of reads (Mbp)	355.7	444.8	298.6	358.6	471.5
Draft genome length (Mbp)	3.0	3.1	3.0	3.0	3.0
Avg coverage (×)	118	145	99	118	155
Avg GC content (%)	37.5	37.5	37.5	37.5	37.5
No. of contigs	23	26	23	27	26
*N*_50_ (bp)	524,550	456,368	442,357	442,262	456,368
No. of protein-coding sequences	2,916	2,996	2,930	2,951	2,969
No. of pseudogenes	18	23	15	20	17
No. of rRNAs	4	3	3	4	4
No. of tRNAs	50	50	59	51	50

The ST14 strains were isolated from blood samples (10 mL) from patients with listeriosis, which were incubated at 37°C for up to 24 h under both aerobic and anaerobic conditions. Then, 0.1 mL of each sample was incubated at 37°C for 24 h on 5% bovine blood agar plates, and one typical colony was subjected to confirmatory biochemical tests for L. monocytogenes. Resistance to arsenic and cadmium was determined as described previously (6). The DNeasy blood and tissue kit (Qiagen, Valencia, CA, USA) was utilized to extract genomic DNA from cells grown overnight at 37°C in brain heart infusion broth (Becton, Dickinson and Co., Sparks, MD, USA). Subsequently, 0.5 to 1 ng of the DNA was used to prepare paired-end libraries with a Nextera XT DNA library preparation kit (Illumina, San Diego, CA, USA). Raw sequencing reads were acquired with either a NextSeq 500 sequencer with the NextSeq 500/550 high-output kit v2.5 (300 cycles, 2 × 150-bp reads) (Illumina) or a MiSeq desktop sequencer with the MiSeq kit v2 (500 cycles, 2 × 250-bp reads) (Illumina). Quality checks, adaptor trimming, and quality filtering utilized CLC Genomics Workbench v7.5.1 (CLC bio, Boston, MA, USA) with default parameters. Reads were assembled with SKESA v2.2 (7), and the resultant contigs were annotated by NCBI Prokaryotic Genome Annotation Pipeline v2021-01-11.build5132 (Table 1) (8). ST and CC designations were assigned via BIGSdb-Lm, hosted by Institut Pasteur (https://bigsdb.pasteur.fr/listeria) (9). LGI2 was identified using Genome Comparator (https://bigsdb.pasteur.fr/cgi-bin/bigsdb/bigsdb.pl?db=pubmlst_listeria_isolates&page=plugin&name=GenomeComparator) and BLAST2 v2.12.0+ (10) comparisons with the LGI2 in serotype 4b strain Scott A. The genome sequence data in this announcement will deepen our understanding of the genetic characteristics of potentially hypervirulent lineage II strains of ST14 (CC14).

### Data availability.

The draft genome sequences and raw sequencing reads were deposited in the GenBank and SRA databases, respectively, under the accession numbers shown in Table 1.
